# Effects of associated SCF and G-CSF on liver injury two weeks after liver damage: A model induced by thioacetamide administration

**Published:** 2014-06

**Authors:** Mohsen Esmaili, Durdi Qujeq, Ali Asghar Yoonesi, Farideh Feizi, Mohammad Ranaee

**Affiliations:** 1 Department of Biochemistry and Biophysics, Faculty of Medicine, Babol University of Medical Sciences, Babol, Iran; 2 Cellular and Molecular Biology Research Center (CMBRC), Babol University of Medical Sciences, Babol, Iran; 3 Department of Anatomical Sciences, Faculty of Medicine, Babol University of Medical Sciences, Babol, Iran; 4 Department of Pathology, Faculty of Medicine, Babol University of Medical Sciences, Babol, Iran

**Keywords:** Granulocyte colony–stimulating factor, Liver injury, Stem cell factor, Thioacetamide

## Abstract

The present study aimed at investigating the beneficial effects of co-administering granulocyte colony–stimulating factor (G-CSF) and stem cell factor (SCF) in a model of chronic liver injury induced by thioacetamide (TAA). Biochemical and histopathology- cal examinations were performed on serum and liver specimens. At the end of the treatment period, the rats were anesthetized with ether, serum was collected and sections of randomly selected fixed liver specimens from each group were embedded in paraffin and processed for light microscopy by staining individual sections with hematoxylin- eosin (HE) stain. Administration of a combination of G-CSF+SCF was carried out two weeks after the TAA treatment. Livers of rats treated with TAA alone exhibited damage, which was significantly less in the group treated with the combination of SCF and G-CSF. Albumin level was 2.35 (g/dl) in the G-CSF+SCF and 1.03 in the TAA- alone group. These differences were statistically significant (P<0.05). Also, in the G- CSF+SCF and TAA group the total protein means (7.16 versus 3.57 mg/dl, respective- ly) were higher than those of the TAA-alone group, and the differences were statistically significant (P<0.05). In the G-CSF+SCF and TAA group the total bilirubin content mean (0.15 versus 0.14 mg/dl, respectively) this difference was not statistically significant (P>0.05).

## INTRODUCTION

Experimental evidence suggests that in liver injury, G-CSF accelerates the regeneration process. Research shows that the granulocyte-colony stimulating factor reduces liver injury. It is widely known that in liver injury models, G-CSF administration could significantly increase the survival rate of rats [1, 2]. Previous studies suggest that granulocyte-colony stimulating factors (G-CSF) reduce liver injury and play a vital role in biology [3]. Many laboratory findings have demonstrated that the combination of G-CSF and stem cell factors (SCF) beginning 3 days prior to myocardial infarction improves cardiac function [4]. The combination of SCF and G-CSF in vivo has also been reported to increase the mobilization of peripheral blood progenitor cells than that seen with G-CSF alone [5]. Investigators have found that Thioacetamide (TAA) is an hpatoxin, in the liver tissue; furthermore, it is metabolized to active and more toxic potent oxide forms such as sulfoxide [6, 7]. There is, in fact a growing body of literature on TAA being a hepatotoxin causing pathologic changes in animal models [8]. Acute administration of TAA in experimental models causes liver lesions [9], but chronic administration of TAA in an animal model can induce liver injury [10]. In this study, we were interested in understanding further mediating effects of SCF+G-CSF on liver damage.

## MATERIALS AND METHODS

**Materials: **Twenty five g TAA T3057, granulocyte colony stimulating factor, human recombinant expressed in E.Coli. (G-CSF), product number G0407, and Gama glutamyltranspepetidase, ALP, 89007 were obtained from Sigma Chemical Co, St Louis, Mo.

**Chronic liver damage model: **Male Wistar rats (150-220 g) were obtained from Babol University Animal Center. The animals were kept in a 12 h light-dark cycle at constant temperature and humidity, and had free access to tap water during the study period. The study was carried out following the guidelines for animal experiments. All animals were carefully maintained under standard animal house conditions. Furthermore, all protocols involving animals were approved by Babol University Animal Care and Use Committee. Effort was made to minimize the number of animals. The approval of the Ethics Committee of Babol University was also obtained (NO: PJ30.3989, 90.3.31). To induce liver injury, rats were given intraperitoneal injections of TAA, 87.5 mg/kg, twice a week for four weeks. Rats were divided into three groups, each group consisting of 7 rats. Group I, the control group, received only saline (5 ml/kg), group II, was the TAA-only treatment group (87.5 mg/kg in 5 ml/kg saline), and group III first received TAA (87.5 mg/kg in 5 ml/kg saline) and then a combination of G-CSF (150 µg/kg) + SCF (50 µg/kg) two weeks after.

**Liver histopathology analysis: **Livers were processed for light microscopy. This consisted of fixing the specimens in 5% neutral formalin solution, embedding them in paraffin, making 5µm thick sections and staining the sections with hematoxylin and eosin.

**Blood Analysis: **Rats were anesthetized and 500µl blood was drawn from the tail vein. Samples were centrifuged at 700 g for 10 minutes and serum was collected. At the start and end of the experimental procedure, biochemical parameters such as blood albumin, total protein and total bilirubin were measured by spectrophotometer after TAA administration.

**Survival rate: **Survival rate was determined four weeks after the first TAA injection in separate groups of rats.

**Statistical analysis: **Statistical analyses were performed using Student's *t*-test and P<0.05 was considered for statistical significance. Results for each treatment are given as mean ± SD of the three groups. SPSS software (version 18.0) was used to analyze the data.

## RESULTS AND DISCUSSION

**Figure 1:**
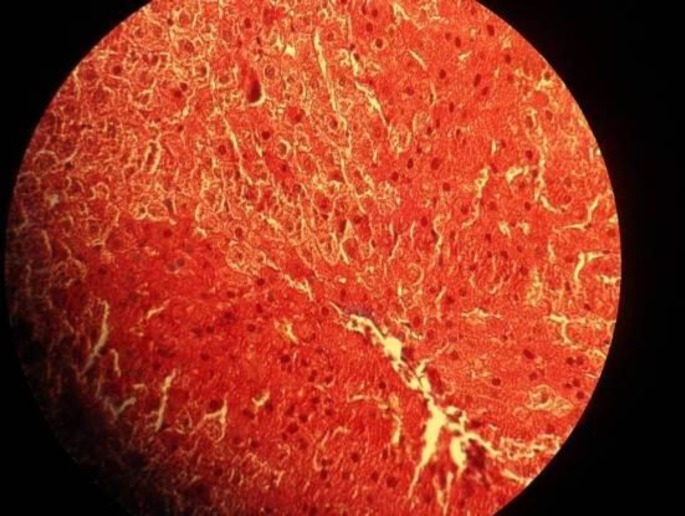
Liver section from a rat that received only normal saline

**Figure 2:**
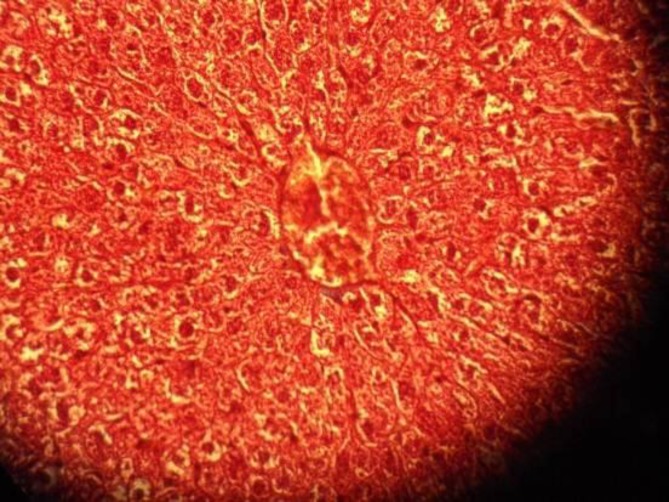
Liver section from a rat that received only TAA

**Figure 3:**
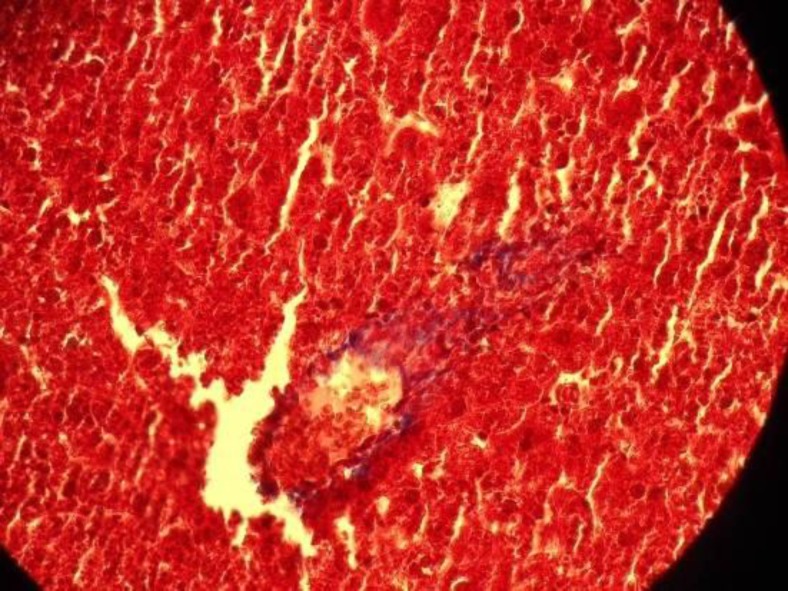
Liver section from a rat that received TAA plus G-CSF+SCF

Significant differences in serum biochemical markers were observed between the control and the TAA treated group. Compared to the control rats, TAA-treated rats had decreased albumin and total protein content (Table 1). In the G-CSF+SCF and TAA group, the mean albumin and total protein content increased. TAA–treated rats showed the lowest decrease in serum levels of total bilirubin (Table 1). Also, in rats treated with TAA, evidence of increased liver damage was observed compared to the control group (Fig. 1 and 2). In rats treated with G-CSF+SCF, demonstrated as a decrease in liver tissue disruption (Fig. 3).

**Table 1:** Serum albumin, total protein and total bilirubin content in control, TAA treated group and TAA plus G-CSF+SCF treated groups

**Biochemical parameters**	**Control group**	**TAA treated group**	**TAA+G-CSF+SCF treated group**
**Serum total bilirubin (mg/dl)**	0.16 ±0.04	0.14±0.02	0.15±0.02
**Serum albumin (gr/l)**	2.31±0.19	1.03±0.12	2.35±0.21
**Serum total protein (gr/l)**	6.27±1.21	3.57±0.89	7.16±1.18

Four weeks after the first TAA injection, the survival rate was significantly higher in groups treated with TAA plus G-CSF+SCF compared to those receiving the TAA only treatment. All rats receiving G-CSF+SCF were alive 16 weeks after the administration of TAA, whereas the TAA only group had a 65% survival rate.

We have reported previously that G-CSF helped repair liver injury [10-12], but in this study by the combined application of SCF and G-CSF liver damage repair significantly increased. Also, in the present study our results indicated that the administration of TAA caused liver injury as demonstrated by the changes of biochemical factors and liver tissue disruption. The occurrence of liver injury may be due to the TAA metabolism which causes the generation of materials leading to cell damage or inflammatory responses to the products of cell lyses. The administration of TAA showed that the serum content of albumin and total protein values decreased. Previous study showed that G-CSF exerts a beneficial effect on the regenerative process in the liver graft by its mobilizing hematopoietic stem cells [13]. A large body of evidence demonstrates that decreased albumin and total protein content is regarded as key factor in the development of liver damage complications [14]. In contrast, the results of the present study indicated that treatment with G-CSF+SCF two weeks after TAA administration caused a marked elevation in the level of serum albumin and total protein and decreased liver tissue disruption. This elevation may be attributed to the liver repair capacity of G-CSF+SCF.

In our study, G-CSF+SCF administration enhanced liver tissue repair, occurring as biochemical and histopathologic reaction after TAA induced liver injury in rats. The mechanism of action of G-CSF+SCF on liver repair remains unclear, and growth factors might exert direct or indirect effects on liver cells through the biochemical and physiological pathways. As previously described, G-CSF alone exerts only a modest effect [10, 11], but in combination of G-CSF and SCF demonstrated better effect [15].

It seems that the combination of SCF and G-CSF caused higher potency and increased repair as compared with G-CSF alone. Our findings are consistent with other studies demonstrating that the combination of SCF plus G-CSF caused a synergistic increase in liver repair as compared with G-CSF alone [16, 17]. These positive effects can be attributed to the stimulated production of growth factors .Another mechanism may be partially and indirectly involved in the biological actions of SCF and G-CSF.

Following partial liver transplants in rats, many investigators have demonstrated that G-CSF administration improves liver regeneration [18]. Others have reported that G- CSF decreased liver enzyme activities and stimulated liver regeneration in chemically- induced liver injuries of animal models [16, 17]. The results of our experiments are in agreement with those reported by investigators regarding chemically-induced liver injuries of other animal models [16, 17]. Nevertheless, the findings of the present study differs from other works [19] in that our results may be related to the difference in the method of G-CSF+SCF administration, the time points of the experiments and the dosage of G-CSF+SCF.

At any rate, few investigations have concerned themselves with the role of G- CSF+SCF in liver repair after chemical liver injury. The mechanism by which the interaction between SCF and G-CSF occurs is yet to be fully understood. Additional studies are required to fully elucidate the exact mechanism and sequence of events by which the protective effects of G-CSF+SCF are induced.
